# Detection of Pathogens in the Gut Microbiome Prior to Bloodstream Infection in Children with Acute Myeloid Leukemia or Mature B-Cell Non-Hodgkin Lymphoma

**DOI:** 10.1093/jpids/piag066

**Published:** 2026-07-28

**Authors:** Juliëtte Schmidt, Maria Miguélez Sánchez, Tom Wolfs, Jop Jans, Jan-Tom van der Bruggen, Andries Budding, Sofia el Manouni el Hassani, Tim de Meij, Hermie Harmsen, Bianca Goemans, Friederike Meyer-Wentrup, Louis Bont, Wim Tissing

**Affiliations:** Princess Máxima Center for Pediatric Oncology, Utrecht, The Netherlands; Inbiome, Amsterdam, The Netherlands; Department of Pediatric Infectious Diseases and Immunology, Wilhelmina Children's Hospital, University Medical Center Utrecht, Utrecht, The Netherlands; Department of Pediatric Infectious Diseases and Immunology, Wilhelmina Children's Hospital, University Medical Center Utrecht, Utrecht, The Netherlands; Department of Medical Microbiology, University Medical Center Utrecht, Utrecht, The Netherlands; Inbiome, Amsterdam, The Netherlands; Department of Pediatric Gastroenterology, Amsterdam University Medical Center, Amsterdam, The Netherlands; Department of Pediatric Gastroenterology, Amsterdam University Medical Center, Amsterdam, The Netherlands; Department of Medical Microbiology and Infection Prevention, University Medical Center Groningen, Groningen, The Netherlands; Princess Máxima Center for Pediatric Oncology, Utrecht, The Netherlands; Princess Máxima Center for Pediatric Oncology, Utrecht, The Netherlands; Department of Pediatric Infectious Diseases and Immunology, Wilhelmina Children's Hospital, University Medical Center Utrecht, Utrecht, The Netherlands; Princess Máxima Center for Pediatric Oncology, Utrecht, The Netherlands; Department of Pediatric Oncology and Hematology, University of Groningen, University Medical Center Groningen, Groningen, The Netherlands

**Keywords:** microbiome, infections, children, leukemia, lymphoma

## Abstract

**Background:**

Bloodstream infections (BSIs) cause significant morbidity and mortality in children with hematological malignancies. The gut may serve as a potential reservoir for BSI-causing bacteria. Therefore, this longitudinal study aimed to assess the abundance of BSI-causing bacterial strains in the gut microbiota prior to BSI.

**Methods:**

This prospective longitudinal cohort study included children (1-18 years) diagnosed with acute myeloid leukemia or mature B-cell non-Hodgkin lymphoma. Fecal samples were collected twice weekly, and additionally whenever a BSI was suspected. Gut microbiota were profiled at species level using the PCR-based assay Molecular Culture.

**Results:**

We recruited 26 children, of whom 16 experienced a total of 31 BSI episodes. In total, 18 out of 38 BSI pathogens (47.4%) could be detected in fecal samples prior to BSI. In the BSI episodes caused by enteric bacteria, 75% of all causative enteric bacteria could be found prior to BSI. Also, bacteria not typically associated with the gut could be detected prior to BSI in feces (*Streptococcus mitis* in 100% of cases, *Staphylococcus epidermidis* in 29% of cases). Furthermore, in fecal samples collected after BSI onset and start of antibiotic treatment, 58% of the BSI pathogens could be detected.

**Conclusions:**

BSI pathogens, both gram-negative and gram-positive, can be detected in the gut microbiome prior to BSI, and an increase in relative abundance is related to BSI. Secondly, BSI pathogens may persist despite BSI-directed intravenous antibiotic therapy, potentially preceding future BSIs.

## INTRODUCTION

Over the past decades, advances in pediatric oncology care^1^^,^^2^ greatly improved the prognosis for children with cancer.^3^^,^^4^ As 1 in 5 deaths in childhood cancer is treatment-related, there is growing attention to complications during anti-cancer treatment. Children with hematological malignancies are especially at risk, with about half of all deaths being treatment-related, predominantly due to infections.^5^ Specifically, bloodstream infections (BSIs) contribute significantly to infection-related morbidity^6^^,^^7^ and mortality.^7^^,^^8^

Disturbance of the intestinal microbiome is a risk factor for BSIs.^9^^,^^10^ Advancements in microbiome research have provided more insights into the crucial role of the gut microbiome in health and disease.^11^^,^^12^ Furthermore, evidence is growing on the involvement of the gut microbiome in chemotherapy-induced toxicities.^13^^,^^14^ The interplay between the gut microbiome and chemotherapeutic toxicity is complex,^15^ involving multiple bidirectional pathways.^14^ Key components are inflammation and mucosal barrier injury caused by chemotherapy.^16^^,^^17^ This is accompanied by a decrease in commensal bacteria, which normally provide protection against pathogen colonization.^16^^,^^18^ Antibiotics, sometimes prescribed prophylactically during chemotherapy,^19^ also contribute to the depletion of commensal microbiota.^18^ The resulting state of intestinal dysbiosis allows potential pathogens to expand and dominate the gastro-intestinal tract. In addition, the physical barrier function of the mucosa may be compromised, enabling translocation of these pathogens into the bloodstream and leading to BSIs.^18^

Studies in adults support the hypothesis of the gut microbiome as a possible reservoir of BSI-causing pathogens. These studies could match BSI isolates with gut microbiome constituents in a subset of BSI cases.^20^^,^^21^ Intestinal domination by the corresponding organism, prior to BSI, was also observed in some adult hematopoietic stem cell transplantation (HSCT) recipients.^22^ Furthermore, adult patients with acute myeloid leukemia (AML) or non-Hodgkin lymphoma (NHL), who developed infectious outcomes, had lower Shannon diversity in fecal samples prior to the start of chemotherapy compared with patients without infections. ^23^^,^^24^ These findings indicate that monitoring of the gut microbiota could potentially be used to predict BSIs. This allows for the application of early microbiome-targeted interventions to treat or prevent infections.^23^^,^^24^

Despite promising results in adults, studies assessing the association between gut dysbiosis and treatment-related symptoms are lacking in the pediatric oncology population.^25^ With regard to BSIs, among 6 pediatric oncology patients, Nycz *et al.* detected several corresponding pathogen genera in fecal samples prior to BSI onset,^26^ while Van de Velde *et al.* observed increasing abundances of the BSI pathogen prior to BSI in a patient with leukemia.^27^ To develop more effective preventive strategies during chemotherapy, existing knowledge gaps on the microbiome in pediatric oncology patients must be addressed.^28^

Therefore, this longitudinal study in children with AML and mature B-cell NHL aimed to assess the relative abundance (RA) of BSI-causing bacterial strains in the gut microbiota prior to BSI.

## MATERIALS AND METHODS

### Study Design and Study Participants

This prospective longitudinal cohort study was conducted at the Princess Máxima Center for Pediatric Oncology (Utrecht, the Netherlands) between March 2022 and December 2024. Patients between 1 and 18 years old and diagnosed with AML or mature B-NHL (with multiagent lymphome malin B (LMB) protocol-based chemotherapy) were eligible to participate, as they have a relatively high incidence of BSI.

Patients were monitored during the second and the third chemotherapy cycle, while the first chemotherapy course was used to obtain informed consent (Supplementary Methods 1). Children with AML were treated according to the NOPHO-DBH AML 2012 protocol,^29^ CHIP-AML22 protocol^30^ (from October 2023) or ML-DS 2018 protocol^31^ (children with trisomy 21). Children diagnosed with mature B-NHL received treatment according to the Inter-B-NHL-Ritux 2010 protocol.^32^ Three patients were treated with similar but slightly different protocols.

The study was approved by the Medical Research Ethics Committee Utrecht (21-267/C) and performed in accordance with the principles of the Declaration of Helsinki and Good Clinical Practice. Informed consent was obtained from parents/legal guardians and from children (≥12 years).

### Data Collection

Patients were monitored for the development of a BSI throughout the full follow-up period (start date chemotherapy course II until start of chemotherapy course IV, which takes approximately 2 months, but length is depending on individual clinical characteristics). Data on febrile episodes and hospital admissions, including clinical symptoms, blood culture results (Supplementary Methods 2), laboratory findings, and medication use were obtained from the electronic medical records, as were data on demographics, cancer treatment, and outcomes.

### Sample Collection

Fecal samples were collected twice a week during chemotherapy courses II and III. A last follow-up sample was collected during the first week of the fourth chemotherapy course. An extra fecal sample was collected when patients experienced fever or were suspected of a BSI. For details on sample storage, see Supplementary Methods 3.

### Microbiota Analyses

To study the gut microbiota composition, the microbiota profiling technique Molecular Culture (inBiome, Amsterdam, the Netherlands) was used.^33^^,^^34^ This technique makes use of the 16S-23S-rDNA interspace (IS) region. Different length polymorphisms of this region are species-specific and can be used to identify and differentiate bacterial species using the Molecular Culture species database (inBiome, Amsterdam, the Netherlands). Furthermore, sequence polymorphisms of 16S rDNA are specific for different bacterial phyla.^33^^,^^34^ Detailed information on the Molecular Culture procedures can be found in Supplementary Methods 4 (sample pre-treatment and DNA extraction) and Supplementary Methods 5 (Molecular Culture microbiota protocol).

### Outcomes

Interpretation of the relevance of a positive blood culture may be challenging. Therefore, we used an independent expert panel consisting of 3 pediatric infectious diseases specialists (all blinded for the microbiota analyses). Experts individually evaluated whether a positive blood culture represented a clinically relevant infection or was assessed as contamination. A BSI was defined as a combination of clinical symptoms suggestive of a BSI and a positive blood culture. In cases of blood cultures positive for coagulase-negative staphylococci, the expert determined, based on clinical judgment and clinical information, whether the blood culture was considered contaminated or represented a true BSI. Final classification of an episode was based on the assessment of the majority of the expert panel (at least 2 out of 3 experts). Cases were defined as patients who experienced ≥1 BSIs during follow-up.

### Statistical Analysis

Gut microbiota composition was assessed using different metrics. For alpha-diversity, the Shannon diversity index in each sample in the period before the first positive blood culture of an individual BSI episode was calculated and visualized over time. Furthermore, for each BSI episode, the RA of the BSI-causing organism(s) was calculated in all samples of the corresponding patient. Also, for each BSI pathogen, detection rates were calculated for different RA thresholds (>0%, ≥10%, ≥30%). Dominance of a species was defined as a RA ≥30%. For each BSI pathogen, RAs in case samples (samples from children with a BSI caused by that pathogen) were compared with those in control samples (samples from children without a BSI caused by that pathogen during study follow-up). The control group for a certain pathogen included therefore both children without any BSI during study follow-up and children with BSIs caused by other pathogens. Relative abundances of BSI pathogens during follow-up were visualized for each BSI episode in an abundance plot that also included antibiotic use. In all plots and in the abundance table, day 0 was defined as the date of the first positive blood culture. Fecal samples collected prior to day 0 were defined as samples prior to BSI onset, and fecal samples collected on or after day 0 as samples after BSI onset.

Molecular Culture analysis was performed using Spotfire (TIBCO, Palo Alto, CA, USA). IBM SPSS Statistics (version 31.0.0.0) was used to analyze descriptive statistics. All other analyses were performed in R software (version 4.4.0), including all data visualizations.

During the preparation of this article, ChatGPT was used to assist with language editing.

## RESULTS

### Patient Characteristics

Twenty-six children collected a total of 253 fecal samples (median 8 [range 1-23] fecal samples per patient). Baseline demographics and clinical characteristics are listed in Table 1 (individual patient-level data is provided in Supplementary Table 1). The median age (range) was 6 (1-16) years, and 19% was female. Most patients were diagnosed with AML (57.7%), followed by Burkitt’s lymphoma (30.8%). During the study period, all patients received routine antibacterial- and antifungal prophylaxis (detailed information in Supplementary Results 1).

**Table 1:** Patient demographics and clinical characteristics (*N* = 26)

Characteristic	All patients *N* = 26 (100%)	Children with ≥1 BSI *N* = 16 (61.5%)	Children without BSI *N* = 10 (38.5%)
**Median age in years (range)**	6 (1-16)	6 (1-16)	7 (2-10)
**Age in years, *N* (%)**			
0-4	10 (38.5)	6 (37.5)	4 (40)
5-9	12 (46.2)	8 (50)	4 (40)
10-14	3 (11.5)	1 (6.3)	2 (20)
15-18	1 (3.8)	1 (6.3)	0
**Sex, *N* (%)**			
Female	5 (19.2)	4 (25)	1 (10)
Male	21 (80.8)	12 (75)	9 (90)
**Diagnosis, *N* (%)**			
AML	15 (57.7)	12 (75)	3 (30)
ML-DS	2 (7.7)	2 (12.5)	0
Burkitt’s lymphoma	8 (30.8)	2 (12.5)	6 (60)
Diffuse large B-cell lymphoma	1 (3.8)	0	1 (10)
**Relapse of malignant disease** ^**a**^ **, *N* (%)**	2 (7.7)	2 (12.5)	0
**Number of stool samples**	253	169	84
**Median number of stool samples per patient (range)**			
During whole follow-up	8 (1-23)	11 (1-23)	7 (5-22)
During second chemotherapy	4 (0-13)	5 (0-13)	3 (0-13)
During third chemotherapy	4 (0-10)	5 (0-10)	4 (1-9)
**Last follow-up sample available, *N* (%)**	11 (42.3)	6 (37.5)	5 (50)
**Antibiotics, *N* (%)**	26 (100)	16 (100)	10 (100)
**Antimycotics, *N* (%)**	26 (100)	16 (100)	10 (100)
**Proton pump inhibitors, *N* (%)**	11 (42.3)	9 (56.3)	2 (20)
**Nasogastric tube feeding, *N* (%)**	21 (80.8)	14 (87.5)	7 (70)
**Total parenteral nutrition, *N* (%)**	13 (50)	10 (62.5)	3 (30)
**Number of febrile episodes assessed**	93	68	25
Classified as BSI episode	31	31	0
**Number of patients with BSI episodes, *N* (%)**	16 (61.5)	16 (100%)	0 (0%)

^a^ Relapsed malignancy at study inclusion.

Abbreviations: AML, acute myeloid leukemia; BSI, bloodstream infection; ML-DS, Myeloid leukemia of Down syndrome

### Pathogens in bloodstream infections

During follow-up, 31 BSI episodes occurred in 16 patients (Table 1), caused by 20 different bacterial species, most of which were gram-positive (Table 2). In total, 14 out of 31 BSI episodes were polymicrobial, with the number of cultured BSI pathogens ranging from 2 to 6 (total 55 individual pathogens) (Table 2). *Staphylococcus epidermidis* was the most commonly identified pathogen (*n* = 15/55, 27.3%) followed by *Streptococcus mitis* (group) (*n* = 9/55, 16.4%) and *Staphylococcus hominis* (n = 5/55, 9.1%). *Leptotrichia* species were the most frequently cultured gram-negative pathogens (3 episodes) (Table 2).

**Table 2:** Bacterial Pathogens Isolated from Positive Blood Cultures During all BSI Episodes (*N* = 31)

Pathogen	Number of episodes ^a^
**Gram-positive species**	
*Enterococcus faecalis*	1
*Enterococcus faecium*	2
*Gemella haemolysans*	1
*Granulicatella adiacens/Granulicatella* species	3
*Kocuria rhizophila*	1
*Paenibacillus* species	1
*Micrococcus luteus*	3
*Rothia mucilaginosa*	2
*Staphylococcus epidermidis*	15
*Staphylococcus haemolyticus*	1
*Staphylococcus hominis*	5
*Staphylococcus petrasii*	1
*Staphylococcus saprophyticus*	1
*Streptococcus mitis (group)*	9
**Gram-negative species**	
*Acinetobacter lwoffii*	1
*Capnocytophaga sputigena*	2
*Escherichia coli ESBL positive*	1
*Klebsiella pneumoniae ESBL positive*	1
*Leptotrichia trevisanii/Leptotrichia* species	3
*Pseudomonas luteola*	1

^a^Number of BSI episodes in which the organism was cultured. As 14 of the 31 BSI episodes were polymicrobial, with 2-6 cultured pathogens per episode, the total number of individual pathogens adds up to 55.

BSI, bloodstream infection

Of the 20 different BSI species as shown in Table 2, 8 species (40%) were not detected by the Molecular Culture assay (indicated as N/D [not detected] in Table 3). These 8 species (accounting for 12 out of the total 55 individual BSI pathogens) were not included in further microbiota analysis, leaving 43 remaining individual pathogens. For 38 of the 43 individual BSI pathogens, fecal samples of the corresponding patient prior to infection were available (Table 3).

**Table 3:** Detection and relative abundances of BSI organisms in fecal samples relative to BSI onset

Patient ID	BSI ID	Result blood culture	Day ^a^ RA ^b^																						Median (IQR) RA in controls ^c^
1	1	*Staphylococcus epidermidis*	Day	−44	−41	−22	−20	−6																	
			RA (%)	0	0	0	0	0																	0 (0-0)
4	2	*Klebsiella pneumoniae ESBL positive*	Day	−48	−45	−40	−35	−32	−29	−28	−24	−22	−20	−7	**−2**	**1**	**5**	**10**	**17**						
			RA (%)	0	0	0	0	0	0	0	0	0	0	0	**33.4**	**40.2**	**27.5**	**12.8**	**28.0**						0 (0-0)
6	3	1. *Granulicatella adiacens*	Day	**−13**																					
			RA (%)	**25.3**																					0 (0-0)
		2. *Streptococcus mitis*	Day	**−13**																					
			RA (%)	**66.5**																					0 (0-4.4)
6	4	*Rothia mucilaginosa*	Day	**−44**																					
			RA (%)	**8.17**																					0 (0-0)
7	5	*Staphylococcus epidermidis*	Day	−13	−10	−7	1	**2**	**6**	**7**	**10**	11	**18**	**22**	**27**	32	37	39	40	46	54	61	62	**75**	
			RA (%)	0	0	0	0	**18.7**	**28.5**	**52.0**	**15.8**	0	**75.3**	**28.5**	**15.0**	0	0	0	0	0	0	0	0	**13.1**	0 (0-0)
7	6	*Rothia mucilaginosa*	Day	−64	−61	−58	−50	−49	−45	−44	−41	−40	−33	−29	**−24**	−19	−14	−12	−11	−5	3	10	11	24	
			RA (%)	0	0	0	0	0	0	0	0	0	0	0	**5.98**	0	0	0	0	0	0	0	0	0	0 (0-0)
8	7	*Staphylococcus hominis*	Day	−48	−40	−23	1																		
			RA (%)	N/D	N/D	N/D	N/D																		N/D
8	8	1. *Micrococcus luteus*	Day	−55	−47	−30	−6																		
			RA (%)	0	0	0	0																		0 (0-0)
		2. *Staphylococcus hominis*	Day	−57	−49	−32	−8																		
			RA (%)	N/D	N/D	N/D	N/D																		N/D
9	9	1. *Enterococcus faecium*	Day	**3**	6	**13**	**14**	**16**	**19**	**25**	**37**	**48**	**52**	**58**	**61**	**65**	**74**	**88**							
			RA (%)	**4.1**	0	**54.6**	**62.3**	**45.9**	**41.6**	**45.0**	**70.3**	**38.9**	**12.1**	**45.5**	**37.0**	**43.0**	**26.6**	**38.4**							0 (0-0)
		2. *Granulicatella adiacens*	Day	**3**	6	13	14	16	19	25	**37**	48	52	**58**	**61**	65	**74**	**88**							
			RA (%)	**8.82**	0	0	0	0	0	0	**3.11**	0	0	**3.30**	**3.24**	0	**2.72**	**11.0**							0 (0-0)
		3. *Staphylococcus epidermidis*	Day	2	5	12	13	15	18	24	36	**47**	51	57	60	64	73	87							
			RA (%)	0	0	0	0	0	0	0	0	**14.4**	0	0	0	0	0	0							0 (0-0)
10	10	*Streptococcus mitis*	Day	**−46**	**−43**	**−36**	**−34**	**−31**	**−13**	**−11**	**−4**	**4**	**6**	**8**	**13**										
			RA (%)	**0.60**	**4.55**	**9.77**	**4.02**	**32.4**	**0.76**	**6.01**	**64.9**	**36.2**	**39.3**	**33.9**	**28.6**										0 (0-4.4)
12	11	*Streptococcus mitis*	Day	−14	−13	**−5**	**2**	**8**	**14**	**16**	**19**	**26**	42	49	**53**	**56**									
			RA (%)	0	0	**0.22**	**0.56**	**2.51**	**0.83**	**0.69**	**19.5**	**23.7**	0	0	**0.34**	**0.31**									0 (0-4.4)
12	12	*Leptotrichia trevisanii*	Day	−53	−52	−44	−37	−31	−25	−23	−20	−13	3	10	14	17									
			RA (%)	0	0	0	0	0	0	0	0	0	0	0	0	0									0 (0-0)
14	13	1. *Staphylococcus epidermidis*	Day	−4	6	9	15	21	34	37	**45**	49	51	53	56	57	61	64	79						
			RA (%)	0	0	0	0	0	0	0	**8.26**	0	0	0	0	0	0	0	0						0 (0-0)
		2. *Pseudomonas luteola*	Day	−4	6	9	15	21	34	37	45	49	51	53	56	57	61	64	79						
			RA (%)	N/D	N/D	N/D	N/D	N/D	N/D	N/D	N/D	N/D	N/D	N/D	N/D	N/D	N/D	N/D	N/D						N/D
14	14	1. *Streptococcus mitis*	Day	**−48**	−38	−35	−29	−23	−10	**−7**	**1**	5	7	9	12	13	17	**20**	35						
			RA (%)	**1.38**	0	0	0	0	0	**1.16**	**8.02**	0	0	0	0	0	0	**1.29**	0						0 (0-4.4)
		2. *Staphylococcus epidermidis*	Day	−55	−45	−42	−36	−30	−17	−14	**−6**	−2	0	2	5	6	10	13	28						
			RA (%)	0	0	0	0	0	0	0	**8.26**	0	0	0	0	0	0	0	0						0 (0-0)
		3. *Staphylococcus saprophyticus*	Day	−55	−45	−42	−36	−30	−17	−14	−6	−2	0	2	5	6	10	13	28						
			RA (%)	N/D	N/D	N/D	N/D	N/D	N/D	N/D	N/D	N/D	N/D	N/D	N/D	N/D	N/D	N/D	N/D						N/D
15	15	1. *Escherichia coli ESBL positive*	Day	**−12**	**−2**	**3**	**5**	**8**	**17**	**20**	**24**	**32**	**37**	**42**	**44**	**57**	**61**	**65**	**70**						
			RA (%)	**37.8**	**26.3**	**40.5**	**29.4**	**28.8**	**33.3**	**32.0**	**28.9**	**28.9**	**16.4**	**50.5**	**28.5**	**27.6**	**19.6**	**29.2**	**30.0**						0 (0-0)
		2. *Staphylococcus epidermidis*	Day	−12	−2	3	5	8	17	20	24	32	37	42	44	57	61	65	70						
			RA (%)	0	0	0	0	0	0	0	0	0	0	0	0	0	0	0	0						0 (0-0)
		3. *Staphylococcus hominis*	Day	−24	−14	−9	−7	−4	5	8	12	20	25	30	32	45	49	53	58						
			RA (%)	N/D	N/D	N/D	N/D	N/D	N/D	N/D	N/D	N/D	N/D	N/D	N/D	N/D	N/D	N/D	N/D						N/D
15	16	*Staphylococcus epidermidis*	Day	−63	−53	−48	−46	−43	−34	−31	−27	−19	−14	−9	−7	6	10	14	19						
			RA (%)	0	0	0	0	0	0	0	0	0	0	0	0	0	0	0	0						0 (0-0)
15	17	1. *Acinetobacter lwoffii*	Day	−79	−69	−64	−62	−59	−50	−47	−43	−35	−30	−25	−23	−10	−6	−2	3						
			RA (%)	N/D	N/D	N/D	N/D	N/D	N/D	N/D	N/D	N/D	N/D	N/D	N/D	N/D	N/D	N/D	N/D						N/D
		2. *Staphylococcus epidermidis*	Day	−83	−73	−68	−66	−63	−54	−51	−47	−39	−34	−29	−27	−14	−10	−6	−1						
			RA (%)	0	0	0	0	0	0	0	0	0	0	0	0	0	0	0	0						0 (0-0)
		3. *Staphylococcus hominis*	Day	−83	−73	−68	−66	−63	−54	−51	−47	−39	−34	−29	−27	−14	−10	−6	−1						
			RA (%)	N/D	N/D	N/D	N/D	N/D	N/D	N/D	N/D	N/D	N/D	N/D	N/D	N/D	N/D	N/D	N/D						N/D
16	18	1. *Leptotrichia trevisanii*	Day	−5	0	6	22																		
			RA (%)	0	0	0	0																		0 (0-0)
		2. *Staphylococcus epidermidis*	Day	−14	**−9**	**−3**	**13**																		
			RA (%)	0	**43.8**	**100**	**45.1**																		0 (0-0)
18	19	*Staphylococcus hominis*	Day	7	9	15	17	26	27																
			RA (%)	N/D	N/D	N/D	N/D	N/D	N/D																N/D
18	20	1. *Leptotrichia species*	Day	−13	−11	**−5**	**−3**	6	7																
			RA (%)	0	0	**3.15**	**0.42**	0	0																0 (0-0)
		2. *Staphylococcus epidermidis*	Day	−15	−13	**−7**	−5	4	5																
			RA (%)	0	0	**3.08**	0	0	0																0 (0-0)
18	21	*Streptococcus mitis group* (likely *Streptococcus oralis*)	Day	−49	−47	**−41**	**−39**	**−30**	**−29**																
			RA (%)	0	0	**5.23**	**17.0**	**0.35**	**8.61**																0 (0-4.4)
21	22	*Staphylococcus epidermidis*	Day	−1	**2**	5	8	12	16	17	20	**27**	**30**												
			RA (%)	0	**2.91**	0	0	0	0	0	0	**4.82**	**17.0**												0 (0-0)
21	23	1. *Streptococcus mitis*	Day	**−15**	**−12**	**−9**	**−6**	**−2**	**2**	**3**	**6**	**13**	**16**												
			RA (%)	**5.29**	**0.40**	**42.8**	**26.5**	**40.4**	**30.3**	**18.9**	**1.02**	**3.40**	**1.53**												0 (0-4.4)
		2. *Staphylococcus petrasii*	Day	−15	−12	−9	−6	−2	2	3	6	13	16												
			RA (%)	N/D	N/D	N/D	N/D	N/D	N/D	N/D	N/D	N/D	N/D												N/D
		3. *Micrococcus luteus*	Day	−21	−18	−15	−12	−8	−4	−3	0	7	10												
			RA (%)	0	0	0	0	0	0	0	0	0	0												0 (0-0)
		4. *Kocuria rhizophila*	Day	−21	−18	−15	−12	−8	−4	−3	0	7	10												
			RA (%)	N/D	N/D	N/D	N/D	N/D	N/D	N/D	N/D	N/D	N/D												N/D
22	24	1. *Streptococcus mitis*	Day	29	51																				
			RA (%)	0	0																				0 (0-4.4)
		2. *Paenibacillus species*	Day	26	48																				
			RA (%)	N/D	N/D																				N/D
22	25	*Capnocytophaga sputigena*	Day	−7	15																				
			RA (%)	0	0																				0 (0-0)
22	26	1. *Capnocytophaga sputigena*	Day	−15	7																				
			RA (%)	0	0																				0 (0-0)
		2. *Staphylococcus haemolyticus*	Day	−15	**7**																				
			RA (%)	0	**15.9**																				0 (0-0)
23	27	1. *Enterococcus faecium*	Day	**−5**	0	**4**	**7**	10	17	19	22	24	27	32	36	38	43	46	49	54					
			RA (%)	**11.9**	0	**10.7**	**29.2**	0	0	0	0	0	0	0	0	0	0	0	0	0					0 (0-0)
		2. *Enterococcus faecalis*	Day	−5	0	**4**	7	10	17	19	22	24	27	32	36	38	43	46	49	54					
			RA (%)	0	0	**39.0**	0	0	0	0	0	0	0	0	0	0	0	0	0	0					0 (0-0)
		3. *Staphylococcus epidermidis*	Day	−5	0	4	7	10	**17**	**19**	**22**	**24**	**27**	**32**	**36**	**38**	**43**	**46**	**49**	**54**					
			RA (%)	0	0	0	0	0	**15.4**	**38.1**	**86.7**	**63.7**	**46.8**	**43.1**	**60.2**	**23.9**	**46.5**	**25.2**	**53.3**	**88.9**					0 (0-0)
		4. *Micrococcus luteus*	Day	−5	0	4	7	10	17	19	22	24	27	32	36	38	43	46	49	54					
			RA (%)	0	0	0	0	0	0	0	0	0	0	0	0	0	0	0	0	0					0 (0-0)
		5. *Streptococcus mitis group* (likely *Streptococcus parasanguinis)*	Day	**−5**	0	4	7	10	17	19	22	24	27	32	36	**38**	**43**	**46**	**49**	**54**					
			RA (%)	**6.26**	0	0	0	0	0	0	0	0	0	0	0	**6.89**	**9.05**	**12.3**	**5.06**	**1.20**					0 (0-4.4)
		6. *Granulicatella species*	Day	−5	0	4	7	10	17	19	22	24	**27**	**32**	**36**	**38**	**43**	**46**	**49**	**54**					
			RA (%)	0	0	0	0	0	0	0	0	0	**0.48**	**4.55**	**39.8**	**19.1**	**23.0**	**7.94**	**15.2**	**7.22**					0 (0-0)
23	28	*Staphylococcus epidermidis*	Day	−32	−27	−23	−20	−17	**−10**	**−8**	**−5**	**−3**	**0**	**5**	**9**	**11**	**16**	**19**	**22**	**27**					
			RA (%)	0	0	0	0	0	**15.4**	**38.1**	**86.7**	**63.7**	**46.8**	**43.1**	**60.2**	**23.9**	**46.5**	**25.2**	**53.3**	**88.9**					0 (0-0)
24	29	*Streptococcus mitis group* (likely *S. oralis*)	Day	**12**	**13**	**18**	**27**	**47**	**52**	**60**	**64**														
			RA (%)	**33.5**	**34.7**	**37.3**	**33.7**	**41.7**	**30.2**	**41.7**	**38.2**														0 (0-4.4)
24	30	1. *Staphylococcus epidermidis*	Day	−22	−21	−16	−7	13	18	26	30														
			RA (%)	0	0	0	0	0	0	0	0														0 (0-0)
		2. *Gemella haemolysans*	Day	−22	−21	−16	−7	13	18	26	30														
			RA (%)	N/D	N/D	N/D	N/D	N/D	N/D	N/D	N/D														N/D
24	31	*Staphylococcus epidermidis*	Day	−35	−34	−29	−20	0	5	13	17														
			RA (%)	0	0	0	0	0	0	0	0														0 (0-0)

^a^Day of fecal sample before (-) of after (+) the first positive blood culture (=day 0). Fecal samples positive for the corresponding BSI isolate are indicated in bold.

^b^Relative abundance in individual fecal samples

^c^Median relative abundance in samples from controls, defined as patients who did not develop a BSI caused by the corresponding BSI isolate during follow-up

*BSI* Bloodstream infection, *IQR* Interquartile range, *N/D* Not detected (species not detected in database), *RA* Relative abundance

### Gut Microbiota Diversity Prior to BSI

The overall Shannon diversity index (based on the phyla Bacteroidetes, FAFV [Firmicutes, Actinobacteria, Fusobacteria, Verrucomicrobia], and Proteobacteria) of fecal samples was measured prior to each BSI episode (Supplementary Figure 1). In general, no decrease in Shannon diversity was observed (no statistics performed), indicating that there was no loss of microbial diversity prior to BSI.

### Presence and Relative Abundance of BSI-Causing Organisms Prior to BSI

**Figure 1:**
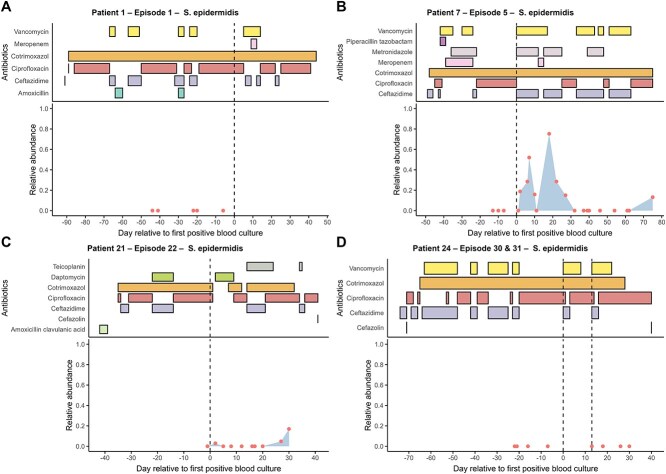
Relative Abundance of *S. epidermidis* in Fecal Samples from Patients Who Experienced One or Multiple BSIs with *S. epidermidis*. The X-Axis Shows Days Relative to the First Positive Blood Culture (=Day 0) of *S. epidermidis*. Dashed Lines Indicate the First Positive Blood Culture for *S. epidermidis* within that Episode. Patient 24 Had 2 BSI Episodes with *S. epidermidis*, Indicated by Two Dashed Lines in the Plot. Horizontal Bars above each Plot Represent the patient’s Antibiotic Use during Study Follow-Up. Panels Represent Individual Patients: (A) Patient 1, Episode 1; (B) Patient 7, Episode 5; (C) Patient 21, Episode 22; (D) Patient 24, Episode 30-31.

**Figure 2:**
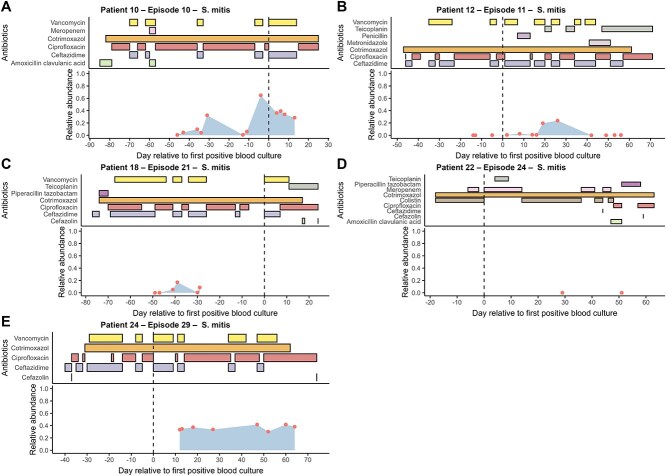
Relative Abundance of *S. mitis* in Fecal Samples from Patients Who Experienced One or Multiple BSIs with *S. mitis*. The X-Axis Shows Days Relative to the First Positive Blood Culture (=Day 0) of *S. mitis*. Dashed Lines Indicate the First Positive Blood Culture for *S. mitis* within that Episode. Horizontal Bars above each Plot Represent the Patient’s Antibiotic Use during Study Follow-Up. Panels Represent Individual Patients: (A) Patient 10, Episode 10; (B) Patient 12, Episode 11; (C) Patient 18, Episode 21; (D) Patient 22, Episode 24; (E) Patient 24, Episode 29.

**Figure 3:**
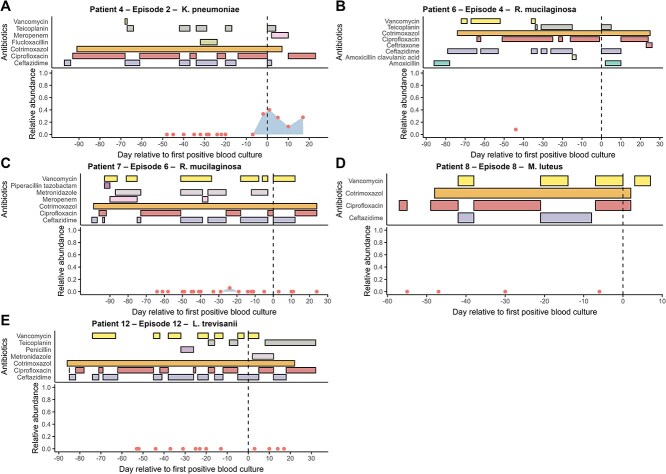
Relative Abundance of BSI Pathogens in Fecal Samples from Patients Who Experienced a Monomicrobial BSI (Other than *S. epidermidis* or *S. mitis*). The X-Axis Shows Days Relative to the First Positive Blood Culture (=Day 0) of that Specific Pathogen. Dashed Lines Indicate the First Positive Blood Culture of a BSI-Species within that Episode. Horizontal Bars above each Plot Represent the Patient’s Antibiotic Use during Study Follow-Up. Panels Represent Individual Patients: (A) Patient 4, Episode 2, *K. pneumoniae*; (B) Patient 6, Episode 4, *R. mucilaginosa*; (C) Patient 7, Episode 6, *R. mucilaginosa*; (D) Patient 8, Episode 8, *Micrococcus luteus*; (E) Patient 12, Episode 12, *Leptotrichia trevisanii.*

**Figure 4:**
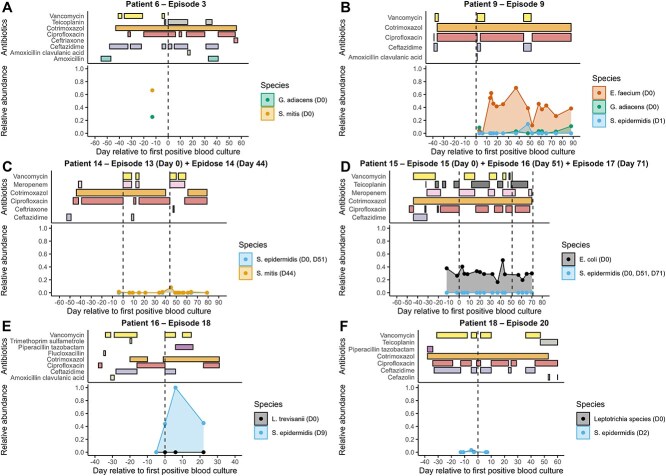

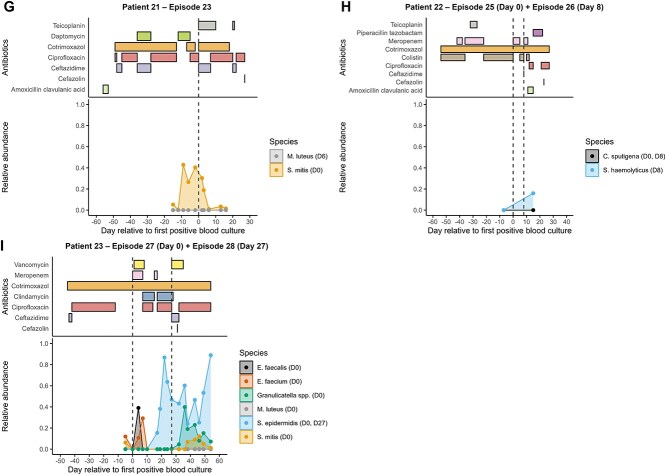
Relative abundance of BSI pathogens in fecal samples from patients with a polymicrobial BSI. The x-axis shows days relative to the first positive blood culture of the first BSI episode (=day 0). When multiple episodes are shown, days are given relative to the first positive blood culture of the first BSI episode (day 0). Dashed lines indicate the first positive blood culture of each BSI episode (multiple lines when multiple episodes are depicted). For each species, the day of its first positive blood culture within the episode (relative to the start of the first BSI episode) is shown in parentheses after the species name. Horizontal bars above each plot represent the patient’s antibiotic use during study follow-up. Panels represent individual patients: (A) Patient 6, Episode 3, *Granulicatella adiacens* and *Streptrococcus mitis*; (B) Patient 9, Episode 9, *Enterococcus faecium, Granulicatella adiacens* and *Staphyloccus epidermidis*; (C) Patient 14, Episode 13-14, *Staphylococcus epidermidis* and *Streptococcus mitis*; (D) Patient 15, Episode 15-17, *Escherichia coli* and *Staphylococcus epidermidis*; (E) Patient 16, Episode 18, *Leptotrichia trevisanii* and *Staphylococcus epidermidis*; (F) Patient 18, Episode 20, *Leptotrichia* species and *Staphylococcus epidermidis*; (G) Patient 21, Episode 23, *Micrococcus luteus* and *Streptococcus mitis*; (H) Patient 22, Episode 25-26, *Capnocytophaga sputigena* and *Staphylococcus haemolyticu*s; (I) Patient 23, Episode 27-28, *Enterococcus faecalis, Enterococcus faecium, Granulicatella* species, *Micrococcus luteus, Staphylococcus epidermidis, Streptococcus mitis*.

An overview of all 31 BSI episodes and RAs of individual BSI pathogens is presented in Table 3. In total, 18 of the 38 individual pathogens (47.4%) could be detected in one or multiple fecal samples prior to BSI (Table 3). For enteric pathogens (*Enterococcus faecalis, Enterococcus faecium, Escherichia coli, Klebsiella pneumoniae*), the percentage that could be detected prior to BSI was 75% (3 out of 4 BSI-pathogens) (Table 3). Both the gram-negative organisms, *E. coli* and *K. pneumoniae,* were highly abundant prior to BSI (RA of 37.8% on day -12 in patient 15, and RA of 33.4% on day -2 in patient 4) (Table 3). Figures 1-4 present a visual overview of the RA in relation to each individual BSI episode, with the top half of the plot showing the antibiotic use of the study participant during follow-up. Figure 1A-D shows *S. epidermidis* BSI episodes, Figure 2A-E shows *S. mitis* BSI episodes, Figure 3A-E shows monomicrobial BSI episodes other than *S. epidermidis* or *S. mitis*, and Figure 4A-I shows polymicrobial BSI episodes. Notably, the RA increased under ciprofloxacin prophylaxis in patient 15 (Figure 4D) and patient 4 (Figure 3A). Antimicrobial susceptibility testing revealed both BSI isolates to be resistant to ciprofloxacin.

Not only enteric bacteria, but also several pathogens that are common colonizers of the oral cavity (*Granulicatella adiacens, Rothia mucilaginosa, S. mitis*) were found in fecal samples prior to BSI (Table 3). *Streptococcus mitis* was isolated from one or more fecal samples in 7 of 7 BSI episodes (100%). Moreover, in 42.9% of the *S. mitis* episodes (3/7), *S. mitis* was a dominant species (Table 3). In patient 6, RA even reached up to 66.5% (Table 3). With regard to other species that are not typically associated with the gut, *S. epidermidis,* a skin commensal, could be detected prior to BSI in 4 of 14 (28.6%) BSI episodes (Table 3). The percentages of pathogens detected in fecal samples at different RA thresholds are shown in Supplementary Table 2.

In samples from controls, the median RA for the individual BSI isolates was 0 (IQR 0-0), except for *S. mitis* (0 [IQR 0-4.4]) (Table 3). To further investigate whether gut domination by BSI-causing species is specific to BSI episodes, it was calculated how frequently these BSI species were detected in samples from control patients at different RA thresholds (Supplementary Table 3). All 12 BSI species could be detected in one or multiple samples from controls during study follow-up. For 6 species, the percentage of controls with the corresponding species was below 10%, for 5 species, the percentage of controls with the corresponding species was between 20 and 30%, and 1 species *(S. mitis)* could be detected in 76.5% of controls. When increasing the RA threshold from >0% to ≥30%, fewer control patients met the detection criteria, and 9 out of 12 species were still detected in controls at the ≥30% threshold. Of these 9 species, 7 species were detected in <9% of controls, and also for the other 2 species, *E. faecium* and *S. mitis,* the number of control patients with the corresponding species decreased to 12.5%, respectively, 23.5%.

### Presence and Relative Abundance of BSI-Causing Organisms after BSI Onset

For 36 of the 43 individual BSI pathogens, one or more fecal samples were available following the positive blood culture (Table 3). In total, 21 out of those 36 BSI-causing organisms (58.3%) could (still) be detected after BSI onset and start of antibiotics (Table 3).

All enteric pathogens (5 of 5) (*E. faecalis, E. faecium, E. coli, K. pneumoniae*) could be detected after BSI onset (Table 3) even as late as day +88 and + 70 in patients 9 and 15, respectively (Table 3, Figure 4B, Figure 4D). These 2 patients did not develop any reinfections during follow-up. However, this was the second *E. coli* BSI for patient 15, as this patient had a prior positive blood culture for *E. coli* before inclusion and sample collection (day -32).

Regarding other bacteria not typically associated with the gut, *S. mitis* could be detected in 6 of 7 episodes (85.7%) after BSI onset in one or multiple samples (dominant in 42.9%) (Table 3). One of the patients (patient 10) with elevated RA levels postinfection developed another positive blood culture with *S. mitis* after study follow-up (day +31). Subsequently, this patient was started on prophylactic teicoplanin intravenously, as this was the third *S. mitis* bacteremia in this patient (first infection on day -62 [prior to start of study], second infection on day 0 [included in this study]). Another patient (patient 12) also had had a first *S. mitis* before study follow-up and received prophylactic teicoplanin following the second infection on day 0 (included in this study).

*Staphylococcus epidermidis* could be detected in 7 of 13 episodes (53.8%) after BSI onset (Table 3). In 3 patients (patients 7, 16, 23 [2 episodes]), the percentage of RA reached ≥30% in one or multiple samples after BSI onset (even up to 88.9% in patient 23) (Table 3). None of these 3 patients developed another positive blood culture with *S. epidermidis* during the study period. However, it should be noted that both patient 7 and 16 had already had a positive blood culture with *S. epidermidis* before study inclusion and sample collection (day -30, respectively day -35). Furthermore, patient 16 was the only patient who developed another positive blood culture for *S. epidermidis* after the study period (day +61).

## DISCUSSION

In this study, BSI pathogens were detected in fecal samples prior to the first positive blood culture and in some cases constituted a large part of the total gut microbiota. Several patients had increased abundances of the BSI pathogen(s) even after antibiotic treatment and clinical recovery, which potentially makes them prone to reinfections with the same micro-organism. The results suggest that the gut acts as a reservoir for BSI-causing pathogens, not only for enteric bacteria, but also for species more commonly associated with the skin/oral cavity, and that part of all BSIs are presumably the consequence of bacterial translocation from the gut. Furthermore, an increase in RA of these species may predict BSIs.

Our results resonate with smaller, pediatric oncology studies that assessed BSI pathogens in the gut microbiota prior to BSI. Kelly *et al.* observed that typical changes in gut colonization preceded most mucosal barrier injury BSIs in pediatric HSCT recipients. Four out of 7 BSIs caused by *E. coli* or *E. faecium* were preceded by gut domination by these strains, and in 2 other BSI episodes, RAs rapidly increased prior to BSI.^35^ In another pediatric HSCT study by Severyn *et al.,* the BSI pathogen was detected in the gut in 7 of the 9 BSI episodes, including *Staphylococcus* species.^36^ Another study in pediatric oncology patients identified 7 of the 11 parental genera of the BSI-causing species.^26^ The results from our larger cohort of BSI cases reinforce the role of the gut as a reservoir for potential BSI pathogens.

Interestingly, in our data, *S. mitis* and *S. epidermidis*, both not typically associated with the gut, were isolated in high abundances in fecal samples before and after BSI onset. In HSCT recipients, non-enteric species *Pseudomonas aeruginosa*^20^ and *S. epidermidis* were also observed in the gut of BSI patients.^20^^,^^36^ Our results, together with evidence from previous studies in the literature, suggests that the gut microbiota can also be a reservoir for BSI pathogens that are not typically associated with the gut, which raises the question whether prophylaxis based on specific gut microbiota composition, rather than standard antibiotic regime, might prevent BSIs. Severyn *et al.* reported a lower cumulative incidence of BSIs in pediatric HSCT recipients who had received oral vancomycin (gram-positive coverage) and polymyxin B (gram-negative coverage), compared to patients without gut decontamination.^36^ Also, in a large randomized clinical trial, prophylactic levofloxacin (gram-positive and gram-negative coverage) lowered the risk of bacteremia in children with leukemia, but not in children undergoing HSCT.^37^ In this last study, the microbiome was not studied, so whether overgrowth in the gut was prevented, or infections were treated early before becoming clinically overt, still remains unclear. In our cohort, institutional standard prophylaxis consisted of daily ciprofloxacin, which has previously been shown to be effective in the prevention of gram-negative BSIs^38^ and is consistent with the clinical practice guideline published by Lehrnbecher *et al.*^19^ This prophylactic use of ciprofloxacin may explain the low number of gram-negative pathogens observed in our cohort, with only 9 of 55 cultured pathogens being gram-negative. Notably, in the 2 patients who developed a BSI caused by gram-negative pathogens of enteric origin, these pathogens were resistant to ciprofloxacin, further supporting the hypothesis that ciprofloxacin may partly explain the lower incidence of gram-negative BSI pathogens in our cohort. Future large interventional studies should investigate whether antibiotic therapy that is guided by RAs of potential pathogenic species reduces these pathogens in the gut, thereby preventing infections. Such an approach would align with the principles of antibiotic stewardship, aiming to use antibiotics more precisely and effectively to optimize patient outcomes and minimize resistance.

In our cohort, multiple patients with increased RAs of *S. mitis* or *S. epidermidis* had (a history of) multiple positive blood cultures with the respective BSI pathogen. This highlights the importance of identifying BSI sources to accurately treat or prevent (re)infections. Monitoring of the microbiome and adaptation of antibiotic treatment may be needed to eradicate the pathogen from the gut to prevent re-infections. Interventional studies in patients where BSI pathogens remain increasingly abundant after clinical recovery could elucidate whether additional, targeted antibiotic therapy after BSI, aiming to reduce the pathogen in the gut, prevents reinfection. Because of the incidence of gram-positive BSI pathogens in our study population, additional antibiotic therapy directed at gram-positive bacteria in the gut could be taken into consideration. As part of another clinical trial in our center, some patients received teicoplanin intravenously, targeting gram-positive bacteria. However, intravenous teicoplanin reaches very low concentrations in the gut, as only a small part is metabolized.^39^ Therefore, targeted antibiotic therapy should specifically be selected for its activity within the gut. For example, oral vancomycin, which is already widely used in the treatment of *Clostridioides difficile* infections,^40^ might be a candidate to prevent re-infections with *S. mitis*.

However, as we also detected BSI-causing species in controls, though mostly in lower abundances, decisions should be based on microbial changes over time, rather than one single sample, to lower “false positives”. In our cohort, all 12 BSI-causing species could also be detected in samples from control patients, and particularly for *S. mitis*, the percentage of patients with one or more fecal samples containing *S. mitis* was high (>75%). Increasing the RA threshold from >0% to ≥30% reduced the number of species detected in control samples and the number of control patients in whom these species were detected. Therefore, rather than relying on a single detection, changes in RA over time should be taken into account when monitoring for the development of a BSI. In addition, we hypothesize that evaluating multiple fecal samples longitudinally may further discriminate between patients who do and do not develop BSIs. Future research should investigate these longitudinal trends in RA and evaluate the positive- and negative predictive value of individual species detected in fecal samples. Based on our data, an increase in RA is a microbiome-related risk factor for BSIs, but it should be noted that other factors can also contribute to this risk, such as the amount of mucosal damage and the prescription of prophylactic antibiotics. Such a microbiome-based approach, also considering the RA of pathogens, could complement *antibiotic stewardship* strategies by enabling more precise and dynamic decision-making for antibiotic therapy, ultimately optimizing treatment while minimizing unnecessary use. Larger studies should investigate the number to treat to prevent (re)infections, to determine whether a microbiome-based intervention is a reliable and effective option.

In contrast to a previous study in pediatric HSCT recipients, which observed a loss of gut microbial diversity in the weeks prior to BSI^35^, we did not observe a decrease in Shannon diversity prior to BSI in our cohort. However, in our study, the number of samples collected prior to BSI was severely limited for several BSI episodes, which precluded any definitive conclusions. In addition, we hypothesize that, due to the nature of Shannon diversity as a composite measure of both evenness and richness, changes in microbial composition preceding a BSI may be better described by a shift from beneficial to potentially pathogenic taxa and may therefore not necessarily result in a decrease in Shannon diversity. Also, as we did not collect samples prior to treatment, but only during chemotherapy courses 2 and 3, the gut microbial composition might already be altered in our population, potentially limiting the detection of differences in Shannon diversity prior to BSI.

When interpreting these findings and discussing future strategies, it is important to take strengths and limitations of this study into account. We included a relatively large cohort of BSI cases and were able to identify bacterial taxa at species level using the validated profiling technique Molecular Culture.^34^ A rapid technique that provides species-level abundances within 5 hours following sampling.^34^ Another strength of our study is its longitudinal design and the intensive fecal sampling schedule. As a downside of the intensive sampling, not all patients were able to collect all samples. Moreover, the study was not designed to study reinfections, so the follow-up time has been too short to draw firm conclusions. In addition, the analyses in this study were limited by the small number of patients. In particular, the number of patients with mature B-NHL was too small to permit meaningful subgroup analyses comparing AML patients and mature B-NHL. Another limitation of this study is the lack of sampling of other ecological niches such as the skin, respiratory tract, and oral cavity. As we couldn’t compare the gut microbiome with microbiome data from other body sites, it is possible that although a certain BSI isolate was highly abundant in the gut, it still originated elsewhere in the body. Future studies should combine frequent stool sampling with the regularly taking of skin-, nasopharyngeal-, and oropharyngeal swabs.

Lastly, as Molecular Culture analysis is based on bacterial profiling rather than whole-genome sequencing, this study did not compare genetic relatedness between BSI pathogens in fecal samples and blood cultures. It is therefore not definite that the strain isolated from the fecal sample is identical to the one from the blood culture. However, we aimed to do the microbiome analyses in a way that would be compatible with clinical practice and deliberately chose a method that could be used as a bedside tool, as Molecular Culture is relatively easy to implement and has high-throughput capacities.^34^ It should be noted that in our study, 8 species (40%) could not be detected using the Molecular Culture method. This may be because these species originated from niches other than the gut, although it may also reflect the lack of reference microbiota profile data for these non-detected species.

In conclusion, this explorative study suggests that the gut may serve as a potential reservoir for possible BSI-causing species, not only for gram-negative-, but also for gram-positive species. In several patients, BSI isolates were detected in increased abundances prior to BSI and sometimes stayed abundant even after antibiotic treatment, potentially preceding reinfections. Our preliminary findings support further investigation into the potential role of microbiota profiling in predicting BSIs in clinical practice. Furthermore, empirical antibiotic therapy guided by the RA of potentially pathogenic species should be investigated in future studies to prevent (re)infections in this severely immunocompromised population.

## Supplementary Material

Supplementary_materials_revision_CLEAN_piag066

## Data Availability

All data generated or analyzed during this study are available from the corresponding author upon reasonable request.
