# Supplementary material for: Detection of Pathogens in the Gut Microbiome Prior to Bloodstream Infection in Children with Acute Myeloid Leukemia or Mature B-Cell Non-Hodgkin Lymphoma
Source: J Pediatric Infect Dis Soc. 2026 Jul 28;15(8):piag066. doi: 10.1093/jpids/piag066 (PMC13464703; doi:10.1093/jpids/piag066)
Supplement: Supplementary file 1 [file supplementary_materials_revision_clean_piag066.docx]

**Supplementary materials**

**Detection of pathogens in the gut microbiome prior to bloodstream infection in children with acute myeloid leukemia or mature B-cell non-Hodgkin lymphoma**

Contents

- Supplementary Methods 1: Chemotherapy protocols
- Supplementary Methods 2: Blood culture
- Supplementary Methods 3: Sample storage
- Supplementary Methods 4: Sample pre-treatment and DNA extraction
- Supplementary Methods 5: Molecular Culture microbiota protocol
- Supplementary Results 1: Antibiotic and antifungal use during study follow-up
- Supplementary Table 1. Demographic and clinical characteristics of individual patients
- Supplementary Table 2. Detection rates of BSI organisms in fecal samples from cases at different relative abundance thresholds
- Supplementary Table 3: Occurrence of increased relative abundances of possible BSI organisms in control patients
- Supplementary Figure 1: Shannon diversity prior to bloodstream infection

**Supplementary Methods 1: Chemotherapy protocols**

Patients were monitored over the course of the second and the third chemotherapy cycle during which clinical data on BSIs and fecal samples were collected. (The time of the first chemotherapy course was needed to obtain informed consent). Children with AML were treated according to the NOPHO-DBH AML 2012 protocol^1^, CHIP-AML22 protocol^2^ (from October 2023) or ML-DS 2018 protocol^3^ (children with trisomy 21). These protocols generally started with a MEC (cytarabine, mitoxantrone, etoposide) course followed by a ADE course (course II, cytarabine, daunorubicin, etoposide) and then a HAM course (course III, cytarabine, mitoxantrone). Etoposide was not included in the ML-DS-18 protocol. Children diagnosed with a lymphoma, received treatment according to the Inter-B-NHL-Ritux 2010 protocol^4^. This consisted of 1 COP course (vincristine, cyclophosphamide, predniso[lo]ne) followed by 2 courses of R-COPADM (course II and III, rituximab, vincristine, predniso[lo]ne, methotrexate, cyclophosphamide, doxorubicin).

**Supplementary Methods 2: Blood culture**

Blood cultures were collected as part of clinical practice during febrile episodes or when a BSI was suspected. As all patients, included in this study, had central venous lines, blood cultures were mostly obtained from central venous lines (199/205 blood cultures). Six blood cultures were obtained peripherally. During blood sampling, a blood culture set was drawn from each lumen of a central venous line. Each blood culture set consisted of 1 aerobic bottle (BD BACTEC Plus Aerobic/F) and 1 anaerobic bottle (BD BACTECT Lytic/10 Anaerobic F). In case of children <4 years of age, a single bottle per lumen was obtained (BD BACTEC Peds Plus/F). All aforementioned blood culture media were produced by Becton, Dickinson and Company (USA). The automated BD BACTEC FX Blood Culture System (Becton, Dickinson and Company, USA) was used for the growth and detection of bacteria from blood specimens. Bottles were typically incubated for 5 days and were considered negative if no growth was detected after these 5 days.

**Supplementary Methods 3: Sample storage**

Sterile plastic containers were used to collect a fecal sample (~2 gram of feces). Fecal samples collected during hospitalization were immediately stored at -20 °C in a freezer at the hospital ward after which they were stored at -80 °C at the laboratory. If patients were at home during the sampling period, they were asked to store their fecal sample within 2 hours in their own freezer (-20 °C). When patients had scheduled hospital appointments, they were requested to transport the samples to the hospital in frozen condition. Upon arrival the samples were immediately stored at -80 °C until further processing.

**Supplementary Methods 4: Sample pre-treatment and DNA extraction**

Frozen fecal samples were thawed on ice and 100 mg of each fecal sample was taken for DNA isolation. After the addition of 200 µL of NucliSens® easyMAG^TM^ lysis buffer, samples were vortexed and incubated at room temperature and 1400 rpm during 5 minutes, followed by centrifugation (18 000 rcf for 2 minutes). Of this, 100 µL of supernatant was used for DNA isolation.

DNA was extracted using an automatic nucleic acid isolation machine; NucliSens® easyMAG^TM^ (bioMérieux). To control for contamination, blank samples were used during DNA extraction. In each well, 100 µL of supernatant was mixed with 2 mL of NucliSens® easyMAG^TM^ lysis buffer. Subsequently samples were incubated at room temperature for 10 minutes and mixed with 70 µL of NucliSens® easyMAG^TM^ Magnetic Silica. For the isolation run, the Specific A protocol was used and an eluate volume of 110 µL Extractionbuffer 3 (BioMérieux NucliSENS® easyMAG™). DNA was stored at 2-8°C.

**Supplementary Methods 5: Molecular Culture microbiota protocol**

To study the gut microbiota composition, the microbiota profiling technique Molecular Culture (inBiome, Amsterdam, the Netherlands) was performed^5,6^. In short, after DNA isolation, the eluted DNA was further diluted (1:10 ratio). Subsequently bacterial DNA was amplified, using two multiplex PCR’s, containing phylum-specific fluorescently labeled primers^5^ that targeted 16S rDNA. The first PCR included primers for Firmicutes/Actinobacteria/Fusobacteria/Verrucomicrobia (FAFV) and Bacteroidetes. The second PCR amplified bacterial DNA of Proteobacteria. Both PCR reactions also contained unlabeled primers specific for 23S rDNA. Furthermore for both PCR reactions a positive control was incorporated (containing bacterial DNA of Firmicutes/Bacteroides or Proteobacteria) to validate the PCR reactions. PCR was carried out with the GeneAMP® PCR System 9700 (Applied Biosystems).

After amplification, 5 µL of the amplified material was mixed with 20 µL of Molecular Culture eMix (inBiome, Amsterdam, the Netherlands) which contained a marker in order to determine IS fragment lengths. The ABI 3500 Genetic Analyzer was used for separation and detection of the IS fragments.

Together, this resulted in peak profiles showing peaks at different IS fragment lengths (numbers of nucleotides) corresponding to specific bacterial species. The Molecular Culture species database (inBiome, Amsterdam, the Netherlands) was used to link fragment lengths to their corresponding bacterial species. The height of a peak indicated the intensity of the fluorescent signal (in relative fluorescence units [RFU]) and corresponded with the abundance of that bacterial species. The color of a peak grouped species into the phyla Firmicutes, Actinobacteria, Fusobacteria and Verrucomicrobia (FAFV)(blue peaks), Bacteriodetes (red peaks) or Proteobacteria (yellow peaks).

**Supplementary Results 1: Antibiotic and antifungal use during study follow-up**

During the study period, all patients received routine antibacterial prophylaxis with ciprofloxacin (30 mg/kg/day in 2 times per day). Additionally, 3 patients with AML received prophylactic teicoplanin (20 mg/kg/once daily three times per week), during the neutropenic period following chemotherapy as part of a clinical trial on the prevention of *Streptococcus mitis* infections. Routine antifungal prophylaxis consisted of itraconazole (5 mg/kg/day once daily) or micafungin (9 mg/kg twice weekly). Furthermore, all patients received trimethoprim/sulfamethoxazole (3/15 - 5/25 mg/kg/day, 3 times per week) as prophylaxis for a *Pneumocystis jiroveci* pneumonia. In case of febrile neutropenia or concern for infection, broad-spectrum antibiotics were started. Standard empirical treatment consisted of both intravenous ceftazidime and vancomycin; however, in some cases alternative antibiotics were prescribed empirically (e.g., due to allergies, carrier status or concomitant medication).

| **Supplementary Table 1. Demographic and clinical characteristics of individual patients** | | | | | | | | | | | |
| --- | --- | --- | --- | --- | --- | --- | --- | --- | --- | --- | --- |
| **Study ID** | **Age** | **Sex** | **Diagnosis** | **Protocol** | **Course 1** | **Course 2** | **Course 3** | **Course 4** | **Antibiotic use** | **Antifungal use** | **BSI Yes/No** |
| 01 | 7 | Male | AML | NOPHO-DBH AML 2012 | MEC | ADE | HAM | HA3E | Amoxicillin  Ceftazidime  Ciprofloxacin  Co-trimoxazole  Meropenem  Vancomycin | Itraconazole | Yes |
| 02 | 6 | Male | Burkitt lymphoma | Inter-B-NHL-Ritux 2010 | COP | R-COPDADM 1 | R-COPADM 2 | R-CYM 1 | Ceftazidime  Ciprofloxacin  Co-trimoxazole  Vancomycin | Itraconazole  Micafungin | No |
| 03 | 4 | Male | AML | NOPHO-DBH AML 2012 | MEC | ADE | HAM | Allo-SCT (conditioning regimen ATG/CloFluBU, GVDH prophylaxis: CsA, MTX) | Ceftazidime  Ciprofloxacin  Co-trimoxazole  Teicoplanin  Vancomycin | Itraconazole  Voriconazole  Amphotericin B | No |
| 04 | 16 | Female | AML | NOPHO-DBH AML 2012 | MEC | ADE + sorafenib | FLA-mylotarg | Allo-SCT (conditioning regimen Clo/Bu/Flu, prophylaxis MTX + CsA) | Ceftazidime  Ciprofloxacin  Co-trimoxazole  Flucloxacillin  Meropenem  Teicoplanin  Vancomycin | Amphotericin B  Isavuconazole  Itraconazole  Micafungin  Voriconazole | Yes |
| 05 | 10 | Male | AML | NOPHO-DBH AML 2012 | MEC | ADE | HAM | HA3E | Ceftazidime  Ciprofloxacin  Co-trimoxazole  Piperacillin/tazobactam  Teicoplanin  Vancomycin | Itraconazole | No |
| 06 | 2 | Male | AML | NOPHO-DBH AML 2012 | MEC | ADE | HAM | HA3E | Amoxicillin  Amoxicillin/clavulanic acid  Ceftazidime  Ceftriaxone  Ciprofloxacin  Co-trimoxazole  Teicoplanin  Vancomycin | Amphotericin B  Itraconazole  Micafungin  Voriconazole | Yes |
| 07 | 6 | Male | AML | NOPHO-DBH AML 2012 | MEC | ADE | HA + mylotarg | HA3E | Ceftazidime  Ciprofloxacin  Co-trimoxazole  Meropenem  Metronidazole  Piperacillin/tazobactam  Vancomycin | Amphotericin B  Itraconazole  Micafungin  Voriconazole | Yes |
| 08 | 7 | Male | Burkitt lymphoma | Inter-B-NHL Ritux 2010 | COP | R-COPDADM 1 | R-COPADM 2 | R-CYVE 1 | Ceftazidime  Ciprofloxacin  Co-trimoxazole  Vancomycin | Amphotericin B  Itraconazole  Micafungin  Voriconazole | Yes |
| 09 | 2 | Male | ML-DS | ML DS 2018 | Vyxeos-1 | Vyxeos-2 | hAM | HA | Amoxicillin/clavulanic acid  Ceftazidime  Ciprofloxacin  Co-trimoxazole  Vancomycin | Itraconazole  Micafungin | Yes |
| 10 | 3 | Male | AML | NOPHO-DBH AML 2012 | MEC | ADE | HAM | HA3E | Amoxicillin/clavulanic acid  Ceftazidime  Ciprofloxacin  Co-trimoxazole  Meropenem  Vancomycin | Itraconazole  Micafungin | Yes |
| 11 | 9 | Male | Burkitt lymphoma | Inter-B-NHL Ritux 2010 | COP | R-COPDADM 1 | R-COPDADM 2 | R-CYVE 1 | Ceftazidime  Ciprofloxacin  Co-trimoxazole  Vancomycin | Micafungin | No |
| 12 | 3 | Male | AML | NOPHO-DBH AML 2012 | MEC | ADE | FLA-DNR -Mylotarg | Allo-SCT (conditioning regimen ATG + CloFluBu) | Ceftazidime  Ciprofloxacin  Co-trimoxazole  Metronidazole  Penicillin  Teicoplanin | Amphotericin B  Isavuconazole  Itraconazole  Micafungin  Voriconazole | Yes |
| 13 | 2 | Male | AML | NOPHO-DBH AML 2012 | MEC | AI (switch to ML DS 2006 protocol) | HAM | HA | Amoxicillin/clavulanic acid  Ceftazidime  Ciprofloxacin  Co-trimoxazole  Metronidazole  Piperacillin/tazobactam  Tobramycin  Vancomycin | Fluconazole  Itraconazole  Micafungin | No |
| 14 | 6 | Female | AML | NOPHO-DBH AML 2012 | MEC | ADE | HAM | HA3E | Ceftazidime  Ceftriaxone  Ciprofloxacin  Co-trimoxazole  Meropenem  Vancomycin | Itraconazole  Mebendazole | Yes |
| 15 | 7 | Male | AML | NOPHO-DBH AML 2012 | MEC | ADE | HAM | HA3E | Ceftazidime  Ciprofloxacin  Co-trimoxazole  Meropenem  Teicoplanin  Vancomycin | Itraconazole  Micafungin | Yes |
| 16 | 11 | Male | Burkitt lymphoma | Inter-B-NHL Ritux 2010 | COP | R-COPDADM 1 | R-COPDADM 2 | R-CYVE 1 | Amoxicillin/clavulanic acid  Ceftazidime  Ciprofloxacin  Co-trimoxazole  Flucloxacillin  Piperacillin/tazobactam  Trimethoprim-sulfametrol  Vancomycin | Itraconazole  Micafungin  Pentamidine | Yes |
| 17 | 10 | Male | Burkitt lymphoma | Inter-B-NHL Ritux 2010 | COP | R-COPDADM 1 | R-COPADM 2 | R-CYM 1 | Ceftriaxone  Ciprofloxacin  Clindamycine  Co-trimoxazole | Itraconazole  Micafungin | No |
| 18 | 3 | Male | AML | CHIP-AML22 | MEC | ADE | FLA + mylotarg + gilterinib | Allo-SCT (conditioning regimen CloFluBU) | Cefazolin  Ceftazidime  Ciprofloxacin  Co-trimoxazole  Piperacillin/tazobactam  Teicoplanin  Vancomycin | Amphotericin B lipid complex  Amphotericin B  Isavuconazole  Itraconazole  Micafungin  Voriconazole | Yes |
| 19 | 3 | Male | Burkitt lymphoma | SKION B-NHL/B-ALL 2008 | COP | COPADM 1 | COPADM 2 | CYM-1 | Amoxicillin/clavulanic acid  Ceftazidime  Ciprofloxacin  Co-trimoxazole  Gentamicine  Vancomycin | Fluconazole  Itraconazole  Micafungin | No |
| 20 | 8 | Female | Burkitt lymphoma | Inter-B-NHL Ritux 2010 | COP | R-COPDADM 1 | R-COPDADM 2 | R-CYM 1 | Ceftazidime  Ciprofloxacin  Co-trimoxazole  Piperacillin/tazobactam  Vancomycin | Itraconazole  Micafungin | No |
| 21 | 5 | Male | AML | Venetoclax APAL2020D (PedAL/EuPAL) study arm A | FLA-GO | FLA | Allo-SCT (conditioning regimen ATG + Bu/Cy/Mel) | - | Amoxicillin/clavulanic acid  Cefazolin  Ceftazidime  Ciprofloxacin  Co-trimoxazole  Daptomycine  Teicoplanin | Amphotericin B  Micafungin | Yes |
| 22 | 9 | Female | AML | Venetoclax APAL2020D (PedAL/EuPAL) study arm B | FLA-GO + venetoclax | FLA + venetoclax | Allo-SCT (conditioning regimen ATG + Clo/Flu/Bu) | - | Amoxicillin/clavulanic acid  Cefazolin  Ceftazidime  Ciprofloxacin  Colistin  Co-trimoxazole  Meropenem  Piperacillin/tazobactam  Teicoplanin | Amphotericin B  Isavuconazole  Micafungin | Yes |
| 23 | 1 | Female | ML-DS | ML-DS 2018 | Vyxeos-1 | Vyxeos-2 | HAM | HA | Cefazolin  Ceftazidime  Ciprofloxacin  Clindamycine  Co-trimoxazole  Meropenem  Vancomycin | Itraconazole | Yes |
| 24 | 7 | Male | AML | CHIP-AML22 | MEC | ADE | HAM + Dasatinib | Allo-SCT (conditioning regimen Bu/Flu/Clo + ATG) | Cefazolin  Ceftazidime  Ciprofloxacin  Co-trimoxazole  Vancomycin | Amphotericin B  Isavuconazole  Itraconazole  Micafungin  Voriconazole | Yes |
| 25 |  |  |  |  |  |  |  |  |  |  |  |
| 26 | 3 | Male | Burkitt lymphoma | Inter-B-NHL Ritux 2010 group B | COP | R-COPADM1 | R-COPADM2 | R-CYM1 | Amoxicillin/clavulanic acid  Cefazolin  Ceftazidime  Ciprofloxacin  Co-trimoxazole  Vancomycin | Itraconazole  Micafungin | No |
| 27 | 9 | Male | DLBCL | Inter-B-NHL Ritux 2010 group B | R-ICE | R-COPADM1 | R-CYM1 | R-CYM2 | Amoxicillin/clavulanic acid  Cefazolin  Ceftriaxone  Ciprofloxacin  Co-trimoxazole | Itraconazole  Micafungin | No |
| *Allo-SCT* Allogeneic stem cell transplantation*,* *DLBCL* Diffuse large B-cell lymphoma, *ML-DS* Myeloid leukemia of Down syndrome. Additional details on chemotherapy protocols are provided in Supplementary Methods 1. | | | | | | | | | | | |

| **Supplementary Table 2. Detection rates of BSI organisms in fecal samples from cases at different relative abundance thresholds** | | | |
| --- | --- | --- | --- |
|  | **RA threshold** | **Number of pathogens^a^ (%)** | |
|  |  | **Prior to BSI** | **After BSI onset** |
| All BSI pathogens | RA >0% | 18/38 (47.4) | 21/36 (58.3) |
|  | RA ≥10% | 10/38 (26.3) | 19 (52.8) |
|  | RA ≥30% | 7/38 (18.4) | 12/36 (33.3) |
| ‘Enteric’ bacteria^b^ | RA >0% | 3/4 (75) | 5/5 (100) |
|  | RA ≥10% | 3/4 (75) | 5/5 (100) |
|  | RA ≥30% | 2/4 (50) | 4/5 (80) |
| *Streptococcus mitis* | RA >0% | 7/7 (100) | 6/7 (85.7) |
|  | RA ≥10% | 4/7 (57.1) | 5/7 (71.4) |
|  | RA ≥30% | 3/7 (42.9) | 3/7 (42.9) |
| *Staphylococcus epidermidis* | RA >0% | 4/14 (28.6) | 7/13 (53.8) |
|  | RA ≥10% | 2/14 (14.3) | 6/13 (46.2) |
|  | RA ≥30% | 2/14 (14.3) | 4/13 (30.8) |
| ^a^Number of pathogens at or above the relative abundance threshold in at least one sample per BSI episode  ^b^Includes *Enterococcus faecium*, *Enterococcus faecalis*, *Escherichia coli*, and *Klebsiella pneumoniae*  *BSI* Bloodstream infection, *RA* Relative abundance | | | |

| **Supplementary Table 3. Occurrence of increased relative abundances of possible BSI organisms in control^a^ patients** | | | |
| --- | --- | --- | --- |
|  | **Number of control patients with ≥ 1 sample^b^ (%)** | | |
|  | **RA >0%** | **RA ≥10%** | **RA ≥30%** |
| *Capnocytophaga sputigena* | 1/25 (4) | 0/25 (0) | 0/25 (0) |
| *Enterococcus faecalis* | 1/25 (4) | 1/25 (4) | 1/25 (4) |
| *Enterococcus faecium* | 5/24 (20.8) | 5/24 (20.8) | 3/24 (12.5) |
| *Escherichia coli* | 7/25 (28) | 6/25 (24) | 2/25 (8) |
| *Granulicatella adiacens* | 6/23 (26.1) | 5/23 (21.7) | 2/23 (8.7) |
| *Klebsiella pneumoniae* | 2/25 (8) | 2/25 (8) | 1/25 (4) |
| *Leptotrichia trevisanii* | 1/23 (4.3) | 1/23 (4.3) | 1/23 (4.3) |
| *Micrococcus luteus* | 1/23 (4.3) | 1/23 (4.3) | 0/23 (0) |
| *Rothia mucilaginosa* | 5/24 (20.8) | 3/24 (12.5) | 0/24 (0) |
| *Staphylococcus epidermidis* | 4/16 (25) | 3/16 (18.8) | 1/16 (6.25) |
| *Staphylococcus haemolyticus* | 2/25 (8) | 2/23 (8) | 2/25 (8) |
| *Streptococcus mitis* | 13/17 (76.5) | 9/17 (52.9) | 4/17 (23.5) |
| ^a^ For each individual BSI-causing species in the case group, a control group was formed in which control patients were defined as patients who did not develop a BSI caused by the corresponding BSI species during follow-up  ^b^ Number of control patients with a relative abundance for the respective species at or above the threshold in ≥ 1 sample during study follow-up  *BSI* Bloodstream infection, *RA* Relative abundance | | | |

**Supplementary Figure 1.** **Shannon diversity prior to bloodstream infection.** Shannon diversity index in fecal samples collected from patients with AML and NHL in the period preceding a BSI. Each plot shows an individual BSI episode with day 0 on the x-axis as the day of the first positive blood culture. Each black dot is a single fecal sample. Dashed lines are indicating the start of the 2^nd^ chemo course (blue line) and 3^rd^ chemo course (red line). For 4 episodes, no fecal samples prior to infection were available. *BSI* Bloodstream infection.

**References**

1.	Tierens, A. *et al.* Mitoxantrone Versus Liposomal Daunorubicin in Induction of Pediatric AML With Risk Stratification Based on Flow Cytometry Measurement of Residual Disease. *Journal of Clinical Oncology* **42**, 2174–2185 (2024).

2.	Kaspers, G. J. L. *et al.* Chip-AML22 Master Protocol: An Open-Label Clinical Trial in Newly Diagnosed Pediatric De Novo Acute Myeloid Leukemia (AML) Patients Including a Linked Phase II Trial with Quizartinib in FLT3-ITD/ NPM1wt Patients - a Study By the NOPHO-DB-SHIP Consortium. *Blood* **142**, 1532 (2023).

3.	Laszig, S. *et al.* CPX-351 in Down syndrome-associated Myeloid Leukemia: Results and Prognostic Factors from the Phase 3 ML-DS 2018 Trial. *Blood* blood.2025030775 (2025) doi:10.1182/blood.2025030775.

4.	Minard-Colin, V. *et al.* Rituximab for High-Risk, Mature B-Cell Non-Hodgkin’s Lymphoma in Children. *New England Journal of Medicine* **382**, 2207–2219 (2020).

5.	Budding, A. E. *et al.* IS‐pro: high‐throughput molecular fingerprinting of the intestinal microbiota. *The FASEB Journal* **24**, 4556–4564 (2010).

6.	De Meij, T. G. J. *et al.* Composition and stability of intestinal microbiota of healthy children within a Dutch population. *FASEB Journal* **30**, 1512–1522 (2016).
